# How cultural factors along with mental health diagnoses influence the treatment of a stroke patient with no previous mental health history: a case report

**DOI:** 10.1192/j.eurpsy.2023.1741

**Published:** 2023-07-19

**Authors:** P. Setién Preciados, E. Arroyo Sánchez, A. Sanz Giancola, I. Romero Gerechter, M. Martín Velasco

**Affiliations:** Psychyatry, Hospital Universitario Príncipe de Asturias, Alcalá de Henares, Spain

## Abstract

**Introduction:**

Mental health awareness campaigns in the past few years have vastly improved how medical professionals treat mental health patients. However, prejudices and ignorance still interfere in medical practice. In this context, with the case presented we can see that even in presumed mental health diagnoses along with cultural factors (race, language…), the best medical assistance is not ensured.

**Objectives:**

Review how different intersectional factors can determine the treatment patients receive at hospitals.

**Methods:**

Presentation of a patient’s case and review of existing literature, in regards to the influence of race, language barriers and mental health diagnoses when attending patients.

**Results:**

The patient is presumed to suffer from a mental health condition after a battery of initial tests with inconclusive results do not demonstrate an organic origin. Instead of continuing with the medical study, the patient is disregarded as psychiatric even though his profile doesn´t fit beforehand of a fictitious or conversion disorder. The fact that there are also cultural factors in play (race and language) probably unconsciously influence how the medical team treats this patient’s case. Cultural social factors persistently present as barriers in clinical practice.

**Conclusions:**

Race, language barriers and mental health diagnoses as well as other intersectional factors do have a great impact in the treatment patients receive. There is yet a lot to do when it comes to educating health professionals if we want to offer the best medical assistance.

**Disclosure of Interest:**

None Declared

